# The *S. pombe* Histone H2A Dioxygenase Ofd2 Regulates Gene Expression during Hypoxia

**DOI:** 10.1371/journal.pone.0029765

**Published:** 2012-01-03

**Authors:** David Lando, Jenny Balmer, Ernest D. Laue, Tony Kouzarides

**Affiliations:** 1 Gurdon Institute and Department of Pathology, University of Cambridge, Cambridge, United Kingdom; 2 Department of Biochemistry, University of Cambridge, Cambridge, United Kingdom; George Mason University, United States of America

## Abstract

Post-translational modification of histone proteins are known to play an important role in regulating chromatin structure. In an effort to find additional histone modifications we set out to screen enzymes of the 2-oxoglutarate and Fe(II)-dependent (2-OG-Fe(II)) dioxygenase family for activity towards histones. Here we show that the *Schizosaccharomyces pombe* 2-OG-Fe(II) dioxygenase domain containing protein-2 (Ofd2) is a histone H2A dioxygenase enzyme. Using a combination of peptide screening and alanine scanning substitution analysis, we identify an HxxLR motif in H2A as a substrate for Ofd2 activity. Transcriptional profiling indicates that Ofd2 regulates the repression of oxidative phosphorylation genes during hypoxic stress. We show that Ofd2 is recruited to the 5′ end of oxidative phosphorylation genes specifically during hypoxia and that it uses its dioxygenase activity to regulate their transcription. Together, these data uncover a novel histone H2A modifying activity involved in the regulation of gene expression during hypoxia.

## Introduction

In all eukaryotic cells DNA is organised into higher order structures called chromatin. The histone octomer is the basic subunit of chromatin and it comprises four core histone proteins (H3, H4, H2A and H2B) around which DNA is wrapped. A remarkable feature of histone proteins is that they are post-translationally modified at many sites and at least eight classes of such histone modifications have been characterised to date. Importantly, it has been shown that these post-translational modifications play critical roles in regulating chromatin associated processes, such as DNA transcription, replication and repair (reviewed in Kouzarides^1^).

**Figure 1:**
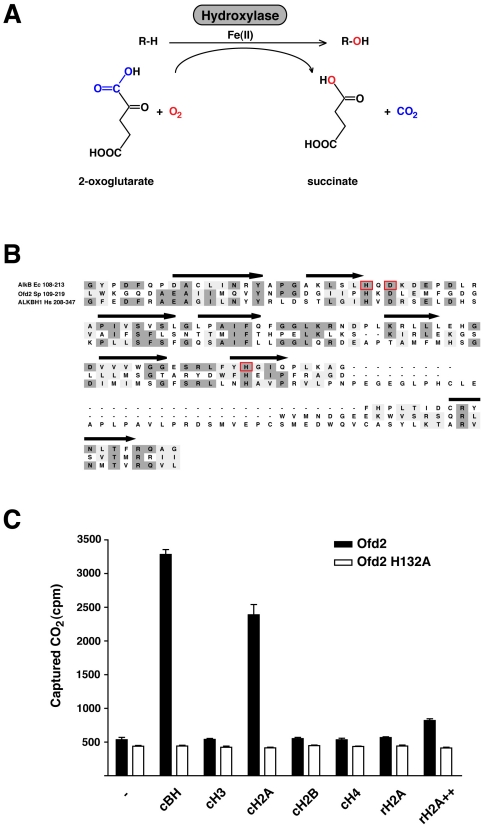
Histone H2A dioxygenase activity. A) General reaction schematic for 2-OG-Fe(II) hydroxylases. R represents an amino acid sidechain. B) ClustalW sequence alignmant of AlkB, Ofd2 and ALKBH1. The secondary structure B-strands for AlkB are dipicted as arrows. Residues in AlkB involved in iron binding are boxed in red. Dark and light shading denote conserved and similar residues respectively. Species abbreviations: Ec *Escherichia.coli*; Sp *Schizosaccharomyces pombe*; Hs *Homo sapiens*. C) Dioxygenase assay of Ofd2 and Ofd2 H132A iron binding mutant. Calf thymus bulk histone (cBH) and individual histones H3, H2A, H2B and H4 and recombinant H2A (rH2A). Dioxygenase activity was evaluated with the CO_2_ capture assay using 1ug of purified Ofd2 with 25ug cBH or 5ug of individual histones, except for ++ where 25ug was used. – indicate control reactions containing no substrates. Data is presented as mean from 2 replicates. Error bars equal 1 standard deviation.

The 2-OG-Fe(II) dioxygenase family of enzymes are widespread in both bacteria and eukaryotes and catalyze a remarkable diversity of reactions, which typically involve the oxidation of a substrate using molecular oxygen (Figure 1A). Among this family are enzymes involved in small molecule biosynthesis including plant hormones and bacterial antibiotics [1], [2], [3]; hydroxylation of amino acid side-chains such as proline and asparagine in hypoxia-inducible factor proteins [4], [5], [6]; oxidative removal of methyl groups from both alkylated nucleic acids and methylated histone proteins [7], [8], [9]; and more recently hydroxylation of 5-methyl cytosine in DNA [10]. Structural studies have revealed that 2-OG-Fe(II) dioxygenases all contain a common ß-strand jelly-roll fold, that is involved in coordinating a catalytically active iron-centre, via a highly conserved HxD/E...H motif [11]. Sequence profile searches have uncovered a large number of proteins, many of which are largely uncharacterised, containing the same jelly roll fold and putative iron centre, typical of 2-OG-Fe(II) dioxygenases [12]. Given the known importance that histone post-translational modifications play in regulating chromatin function, we decided to conduct a screen of theses dioxygenase enzymes for activity with histone proteins.

## Results

### Ofd2 possess histone H2A hydroxylase activity

To search for potential new dioxygenase enzymes that modify histones we employed a radiolabeled CO_2_ capture assay initially developed for collagen proline and lysine hydroxylases [13]. In this assay candidate dioxygenase enzymes were bacterially expressed and then incubated with calf thymus bulk histones (cBH) along with [^14^C]-2-OG and the release of ^14^CO_2_ was monitored. Using this assay we found that when the *S. pombe* protein Ofd2 was incubated with cBH a substantial increase in CO_2_ levels was detected, when compared to no substrate control (Figure 1C). Sequence profile searches have revealed that Ofd2 belongs to the AlkB like sub-family of 2-OG-Fe(II) dioxygenases [12] (Figure 1B). The residues involved in iron binding are well conserved and have been shown to be essential for activity of AlkB like enzymes [14]. To test if the dioxygenase domain of Ofd2 is required for activity we mutated one of the iron coordinating histidine residues to an alanine (Ofd2 H132A) and tested it for activity with cBH (Figure 1C). The catalytic mutant Ofd2 H132A totally abolished release of CO_2_ to background levels, confirming that the dioxygenase domain was required for activity. The cBH comprise the four core histones H3, H2A, H2B and H4. To determine which of these core histones may be the target for Ofd2 we assayed individually purified calf thymus histones with the CO_2_ capture assay and found that the activity was associated with cH2A (Figure 1C). When we assayed Ofd2 with recombinant H2A (rH2A), increased release of CO_2_ was detected – however, unlike cH2A much more rH2A was required in the reaction (Figure 1C). Histone H2A purified from calf thymus is known to contain many different types of postranslational modifications, such as acetylation and methylation of lysine residues [31]. The increase in CO_2_ detected with cH2A over recombinant H2A may indicate that Ofd2 targets an existing modified residue on cH2A or requires a modified residue to recognise its substrate more efficiently.

Of all the human AlkB like homologs Ofd2 is closely related to Alkbh1 (Figure 1B). To test if Alkbh1 also contains histone dioxygenase activity we tested its activity with the CO_2_ capture assay and found that when incubated with calf thymus bulk histones (cBH) a substantial increase in CO_2_ levels was detected, when compared to no substrate control (Fig. S1). Like Ofd2, when we assayed individually purified calf thymus histones for activity with the CO_2_ capture assay we found that Alkbh1 activity was also associated with cH2A. When we mutated one of the iron binding histidine residues to an alanine (Alkbh1 H228A) and tested it for activity with cBH and cH2A the iron binding mutant totally abolished release of CO_2_ to background levels, confirming that the dioxygenase domain is required for activity. Taken together these results suggest that both Ofd2 and Alkbh1 possess dioxygenase activity towards histone H2A.

### Identification of a H2A motif required for Ofd2 dioxygenase activity

**Figure 2:**
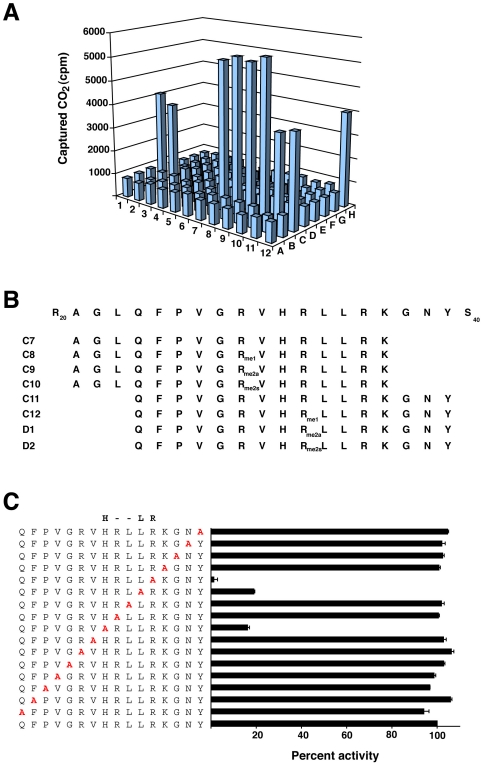
Identification of a motif in H2A required for Ofd2 dioxygenase activity. A) Dioxygenase assay of Ofd2 with H2A peptide library. Activity was evaluated with the CO_2_ capture assay using 1ug of purified Ofd2 with 6ug of peptide or 25ug of BSA (H11) and cBH (H12). For a list of peptide sequences see Table S7. B) Alignment of peptide amino acid sequence of 8 postive peptides (C7-12, D1-2) from A with histone H2A sequence. C) A HxxLR substrate motif for Ofd2. Alanine scanning analysis of peptide sequence C11 was evaluated with the CO_2_ capture assay using 1ug of Ofd2 with 6ug of peptide. Alanine substitutions are indicated in bold red. Data is from 2 replicates presented as a percent activity of captured CO_2_ relative to unmodified C11 peptide. Error bars equal 1 standard deviation. Residues found to be essential for activity are highlighted above peptide sequence.

We decided to identify the H2A substrate for Ofd2 using a peptide library approach and a library comprising of 16 mer peptides derived from H2A was synthesized (Table S7). To date the removal of methylated residues in histones has been shown to be carried out by two distinct classes of enzyme. The LSD1 (Lysine specific demethylase 1) family was the first to be identified and can remove methylation from lysine residues by an oxidative reaction which uses flavin as a cofactor [15]. The second family contains a Jumonji C (JmjC) domain and can remove methyl groups from both lysine and arginine residues via a 2-OG-Fe(II) dependent process [9], [16]. To investigate if Ofd2 could potentially be a histone demethylase we included in our peptide library all possible combinations of methylated lysine and arginine residues, along with unmodified peptides. This library consisting of 94 peptides was then tested with Ofd2 using the CO_2_ capture assay. Using this library we found that Ofd2 activity, as indicated by increase in captured CO_2_, was detected in 8 peptides (C7-C12, D1-D2) out of the 94 peptides assayed (Figure 2A). A closer examination of the 8 peptide sequences found with Ofd2 showed that they all mapped to a common region within H2A, and were comprised of two 16 mer peptide backbones – 3 peptides contained methyl-arginine and 1 was un-methylated (Figure 2B). Interestingly, levels of captured CO_2_ were found to be similar for both the methylated and un-methylated peptides within each peptide backbone, suggesting that arginine methylation was neither required for, nor a substrate of, Ofd2. In an effort to identify the site of modification we carried out mass spectrometry analysis (as described in material and methods). However, we could not detect any mass change on any of the 8 peptides after Ofd2 treatment (data not shown). Currently it is unclear why Ofd2 dioxygenase activity cannot be detected by mass spectrometry. One explanation could be that the dioxygenase activity with the peptides is primarily uncoupled. In this scenario very little or no product would be formed, because conversion of 2-OG to succinate and CO_2_ occur without substrate oxidation. Analysis of other 2-OG-Fe(II) dioxygenases, such as collagen prolyl-4-hydroxylase [17] and *E.coli* AlkB [18], have also reported uncoupled turnover. In these cases uncoupled turnover has been suggested to be due to improper binding of the dioxygenase enzyme to its substrate. It may also be that Ofd2 requires an additional post-translational modification on H2A to correctly bind to its substrate, which may indicate why Ofd2 is significantly more active on cBH, then rH2A.

To gain further insight into which residues were responsible for Ofd2 dioxygenase activity we performed alanine scanning substitutional analysis on peptide sequence C11 (Figure 2C). We found that substitution of 13 of the 16 residues with alanine showed similar percent activities to wild type C11 peptide sequence, indicating that these residues were not essential for Ofd2 activity. However, 3 residues did show significantly lower activity when substituted with alanine. Histidine 7 and leucine 10 substitution reduced activity to approximately 20 percent of wild type, while arginine 11 was reduced to near zero. The reduction in activity suggest that these 3 residues are required for Ofd2 activity. A similar substitutional scanning analysis conducted with peptide C7 gave exactly the same result (Figure S2). Together, our data uncover HxxLR as the minimal motif in H2A required for Ofd2 activity.

### Increased hypoxic repression of oxidative phosphorylation genes in Ofd2 deletion strain

To gain further insight into the function of histone H2A dioxygenase activity we investigated the biological role of Ofd2. Previous work has shown that another *S. pombe* 2-OG-Fe(II) dioxygenase called Ofd1 is involved in the oxygen dependent regulation of Sre1 protein levels in response to hypoxic stress [19]. Interestingly, like Ofd1, the *Ofd2* gene had been shown to be transcriptionally up-regulated by hypoxic stress [20] and we therefore reasoned that Ofd2 might also be involved in the regulation of hypoxia genes. To investigate this we created a deletion strain of Ofd2 (Ofd2**Δ**) and used transcriptional profiling microarray analysis to compare transcriptional profiles in wild type (wt) and Ofd2**Δ** cells before and after 90 min hypoxia treatment. The results can be found in Table S1. To identify potential genes whose expression might be different between the two strains we calculated the change in gene expression in hypoxic compared to normal oxygen conditions (Table S1 +/- hypoxia), and plotted these ratios as log2 values on a scatter plot (Figure S3). Using this procedure we identified in total 25 genes whose expression were increased (green squares) and 42 genes whose expression were reduced (red squares) two fold or greater. Analysis of these genes revealed that some of the genes previously identified as being induced or repressed by low oxygen stress [20], were also induced or repressed in our study (see Tables S2, S3, S4, S5). We applied a threshold limit of 1.5 fold to the gene expression ratios of both the induced and repressed genes to identify those responding differently in the wt or Ofd2**Δ** strains (Figure S1 grey line). We reasoned that any genes lying within this threshold limit would be considered to have similar expression between the two strains, while any lying outside could be considered to be expressed differently. For genes induced in low oxygen conditions, applying this threshold limit revealed that all lay within this threshold. Thus, we conclude that the induction of these genes occurs at a relatively similar level in both the wt and Ofd2**Δ** strains. However, a similar analysis of the repressed genes revealed that 8 out of the 42 lay outside the threshold limit (Figure S1 shaded box). Interestingly, all 8 genes were more repressed in the deletion strain (closer to the Ofd2**Δ** axis). A closer analysis of these 8 genes reveals that 6 out of the 8 are involved in mitochondrial electron transport and ATP synthesis – processes that are associated with cellular energy production via oxidative phosphorylation (Table 1).

**Table 1:** Significantly^a^ hypoxia repressed genes in Ofd2Δ.

Functional category and gene	Name	Description	Fold change +/−hypoxia^b^	
			wt	Ofd2Δ
Oxidative phosphorylation				
SPCC191.07	*cyc1*	cytochrome c	0.24	0.15
SPAC3A11.07	*-*	NADH dehydrogenase	0.33	0.17
SPBC13E7.04	*atp16*	F1-ATPase delta subunit	0.47	0.30
SPAC1782.07	*qcr8*	ubiquinol-cytochrome-c reductase complex subunit 7	0.55	0.35
SPBP4H10.08	*qcr10*	ubiquinol-cytochrome-c reductase complex subunit	0.60	0.36
SPCC613.10	*qcr2*	ubiquinol-cytochrome-c reductase complex core protein	0.63	0.41
Cellular iron ion homeostasis				
SPBC4F6.09	*str1*	siderophore-iron transporter	0.51	0.30
Mitochondrial translation				
SPBC409.22c	*-*	mitochondrial translation elongation factor G	0.70	0.44

a Threshold of 1.5 fold (see Figure S1).

b Presented as average change in gene expression in hypoxia over expression in normal oxygen conditions.

**Figure 3:**
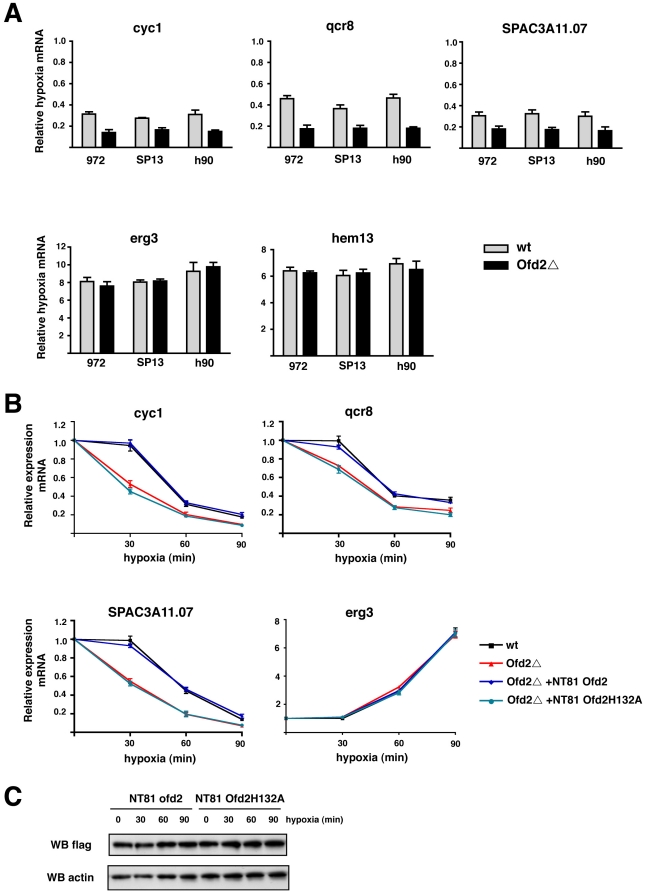
Role for Ofd2 dioxygenase domain in oxygen sensing. A) Three yeast strains (972, SP13 & h90) that were wt and deleted for Ofd2 (Ofd2**Δ**) were cultured for 90 min at both normal oxygen and hypoxia conditions. Total RNA samples were then prepared and mRNA levels of 3 repressed genes (*cyc1, qcr8 & SPAC3A11.07*) and two induced genes (*erg3 & hem13*) were quantitated by RT-qPCR. Expression levels were then normalised to *act1* and the fold change in expression levels of hypoxia over normal oxygen conditions were plotted as relative hypoxia mRNA. Data are the mean from 3 replicate qPCR. Error bars equal 1 standard deviation. B) Hypoxia time course analysis of mRNA levels for 3 repressed and 1 induced gene in wild type (wt), Ofd2**Δ**, Ofd2**Δ** rescued (Ofd2**Δ**+NT81 Ofd2) and iron binding mutant rescued (Ofd2**Δ**+NT81 Ofd2H132A) strains. At 0, 30, 60 and 90 min after hypoxia treatment total RNA samples were prepared and mRNA levels were quantitated by RT-qPCR and normalised to *act1*. Expression levels at each time point were then plotted relative to the levels at time 0. Data are the mean from 2 replicate qPCR. Error bars equal 1 standard deviation. B) Western blot analysis of 25 µg of whole cell extracts of NT81 Ofd2 and NT81 Ofd2H132A strains at various time points after hypoxia treatment with anti-flag and anti actin antibodies.

To confirm our microarray observations we performed reverse transcriptase and quantitative real-time polymerase chain reaction (RT-qPCR) analysis on three oxidative phosphorylation genes (*cyc1, qcr8 and SPAC3A11.07)* repressed during hypoxia and two control genes (*erg3* and *hem1)* which are induced (Figure 3A). The results confirmed our microarray data. The repressed genes *cyc1, qcr8 and SPAC3A11.07* displayed greater repression in the Ofd2**Δ** strains when compared to wt. In contrast, no difference was observed between wt and Ofd2**Δ** strains for the induced genes *erg3* and *hem13*. Analysis of two additional Ofd2**Δ** strains (SP13 and h90) showed similar results. Together with the transcriptional profiling microarray analysis we conclude that in *S. pombe* certain genes associated with oxidative phosphorylation are more repressed during hypoxia treatment when Ofd2 is deleted.

### Oxygen sensing role for Ofd2 dioxygenase domain

To investigate further the role of Ofd2 in regulating hypoxic gene expression we performed a time course experiment with wild type and Ofd2Δ cells, where samples were collected 30, 60 and 90 mins after hypoxic treatment and mRNA levels for selected genes were quantified by RT-qPCR (Figure 3B). In both wild type (black line) and Ofd2Δ cells (red line) we observed relatively little difference in mRNA levels for the induced (control) gene *erg3*, at all time points after hypoxia treatment. This is in agreement with our previous data, which showed no involvement of Ofd2 in affecting hypoxia induced genes. For the oxidative phosphorylation genes, *cyc1, qcr8* and *SPAC3A11.07*, we observed a greater reduction in mRNA expression of these genes at all time points in the Ofd2Δ strain. More interestingly, 30 min after hypoxic treatment, where relatively little or no reduced expression is seen in the wt strain, a markedly greater reduced expression of these genes is observed in the Ofd2Δ strain, suggesting that the Ofd2Δ strain is more sensitive to hypoxic stress. Importantly, all these observations could be rescued when a cDNA for Ofd2 was reintroduced back into Ofd2Δ (Ofd2Δ +NT81 Ofd2), confirming that this effect is specific to the Ofd2 gene.

To test if the H2A dioxygenase domain of Ofd2 is required for Ofd2 activity we mutated the iron coordinating His residues to an Ala (Ofd2H132A) and reintroduced this back into the Ofd2Δ strain and re-tested hypoxia-induced gene expression (Figure 3B). We found that like the Ofd2Δ strain (red line), the Ofd2H132A mutant (purple line) mirrored both the early and reduced expression of mRNA levels for *cyc1, qcr8* and *SPAC3A11.07*. The effect of the mutation was not due to aberrant protein stability as similar protein levels were detected to that of non-mutated Ofd2 (Figure 3C). Collectively, these results suggest that during early exposure of cells to hypoxia, Ofd2 may play a role in the sensing process, inhibiting mRNA reduction until critical levels of oxygen are reached and that this requires the activity of the Ofd2 dioxygenase domain.

### Hypoxia dependant localisation of Ofd2 to repressed genes

**Figure 4:**
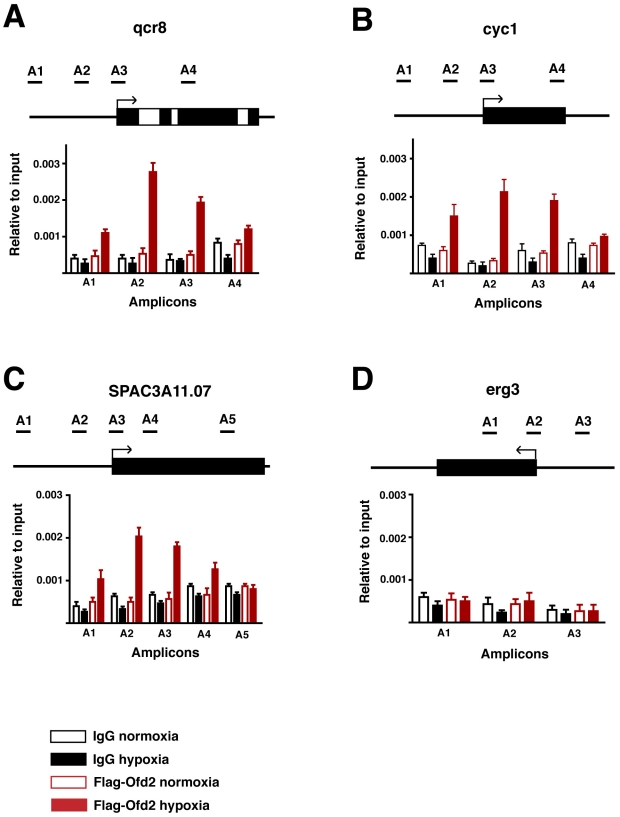
Ofd2 localises to repressed genes during hypoxia. A strain containing flag tagged Ofd2 was grown under hypoxic or normal oxygen conditions for 60 min. Chromatin immunoperciitation was then performed using anti-flag or anti mouse IgG as indicated. Binding to various amplicons in 3 repressed genes. (A–C) and 1 induced gene (D) was assayed using qPCR. Bound DNA is plotted relative to input DNA. Data are the mean from 2 replicate qPCRs. Error bars equal 1 standard deviation. Location of amplicons in each gene is depicted above each graph and arrows indicate 5′ end of gene.

Our finding that Ofd2 possesses dioxygenase activity towards histone H2A raised the possibility that Ofd2 may directly regulate transcription. One way that transcriptional regulators influence transcription is via binding to their chromatin targets [21]. To test if Ofd2 exerts its effect in this way we assayed Ofd2 binding to oxidative phosphorylation genes using chromatin immunoprecipitation (ChIP). Cells containing flag-tagged Ofd2 were cultured in the presence or absence of oxygen for 60 min and Ofd2 immunoprecipitated DNA was quantified using qPCR. When compared to a control immunoprecipitation (IgG), Ofd2 was found to be enriched at the 5′ region of each of the three oxidative phosphorylation genes previously identified as Ofd2 regulated (Figure 4A–C). For each gene, binding of Ofd2 was dependant on hypoxia, as cells cultured in normal oxygen conditions did not ChIP significant amounts above the control IgG. In addition, and in agreement with our previous observations that Ofd2 is not involved in the regulation of induced genes, Ofd2 was not found to be enriched at the hypoxically induced *erg3* gene (Figure 4D). Together, these data suggest that Ofd2 acts directly at the 5′ region of the oxidative phosphorylation genes to regulate their repression in response to low oxygen stress.

## Discussion

Our screen for histone dioxygenase enzymes has uncovered Ofd2 as a novel histone H2A dioxygenase enzyme. By using a combination of peptide screening and alanine scanning substitution analysis we have found an HxxLR motif in H2A that is a substrate for Ofd2. Using transcriptional profiling microarray analysis we then went on to show that Ofd2 is involved in regulating the repression of oxidative phosphorylation genes under hypoxia conditions. We also demonstrate that in cells either deleted for Ofd2, or containing a catalytically inactive Ofd2 dioxygenase domain, the decrease of expression levels of oxidative phosphorylation genes is more rapid, when challenged with hypoxia. Thus, our results identify Ofd2 as a novel histone H2A dioxygenase enzyme involved in the regulation of gene expression during hypoxia.

The exact modification of H2A by Ofd2 remains to be determined. Previous members of the 2-OG-Fe(II) dioxygenase family have been shown to act on protein substrates and to catalyse both hydroxylation of amino acids, such as proline [22] and lysine [23] in collagen, as well as to demethylate methylated amino acids, such as methyllysine in histone proteins [9]. Our analysis did not find evidence of demethylation occuring in our peptide library screen. Although, we cannot rule out the possibility that in certain other circumstances, Ofd2 may catalyse a demethylation reaction, these data suggest that Ofd2 catalyses hydroxylation of an amino acid residue. The HxxLR motif that we identified most likely contains the site of hydroxylation. Interestingly, out of these three amino acid residues, hydroxylation of arginine has been described in proteins extracted from the adhesive plaque and foot of marine mussels [24].

Finally, we show that Alkbh1, a mammalian protein closely related to Ofd2, is also a histone H2A dioxygenase enzyme. In a gene deletion study by Pan et al., [25] *Alkbh1^-/-^* mice displayed impaired placental trophoblast lineage differentiation. Alkbh1 was shown to interact with Mrj, an essential placental protein that recruits class II histone deacetylases to repress transcription during placental development. Therefore, it would seem that H2A dioxygenase activity, catalysed by Ofd2 and Alkbh1, share a common functional role in regulating gene expression.

## Methods

### Strains and plasmids

The following S. pombe strains were used: 972 (h-), SP13 (h- leu1-32), h90 (leu1-32 ade6-210 ura4-D18), DL1003 (h- Ofd2ΔKAN), DL1016 (h-90 leu1-32 ade6-210 ura4-D18 Ofd2ΔKAN), DL1020 (h-, leu1-32, Ofd2ΔKAN), DL1023 (h-, leu1-32 Ofd2ΔKAN ::leu1^+^pREPNT81-Flag-Ofd2), DL1024 (h- leu1-32 Ofd2ΔKAN ::leu1^+^ pREPNT81-Flag-Ofd2H132A). Standard methods and techiques for fission yeast genetic manipulations were employed [26]. Plasmids containing the Ofd2 gene were generated by inserting genomic fragments generated by PCR into pREPNT81 [27] and pET30a (Novagen) to generate N-terminal flag tagged Ofd2 (pREPNT81-Flag-Ofd2) and hexa-histidine tagged Ofd2 (pET30a-His-Ofd2) respectively. The alkbh1 plasmid pET15b-His-ABH1 has been previously described [28]. Mutation of histidine 132 in Ofd2 and 228 in Alkbh1to alanine was carried out using the QuickChange site-directed mutagenesis kit (Stratagene) to generate plasmids pREPNT81-Flag-Ofd2H132A, pET30a-His-Ofd2H132A and -His-ABH1 H228A. All cloning and mutagenesis were verified by sequencing.

### Protein expression and purification

Expression plasmids encoding hexa-histidine tagged full length and iron binding mutant Ofd2 and alkbh1 were transformed into BL21 Codon plus (Statagene). To induce expression, cultures were grown at 37°C to OD_600_ = 0.6 and IPTG was added to a final concentration of 0.5 mM and incubation continued for 1 hr. Harvested cells were then broken by French press in lysis buffer (25 mM Tris-HCl pH 8.0, 300 mM NaCl, 0.1% Triton-X-100, 0.5 mM ß-mercaptoethanol, 5 mM imidazole, protease inhibitors). Lysates were clarified by centrifugation at 12,000×g for 20 min at 4°C, then loaded onto a Ni-NTA agarose column (Qiagen). The resin was washed 3× with 20 column volumes of wash buffer (25 mM Tris-HCl pH 8.0, 300 mM NaCl, 0.5 mM ß-mercaptoethanol, 20 mM imidazole). Bound proteins were then eluted in wash buffer containing 250 mM imidazole. Purified proteins were then dialysed into storage buffer (25 mM Tris-HCl pH 8.0, 150 mM NaCl, 0.5 mM DTT, 5% glycerol).

### Histone proteins and peptides

Calf thymus bulk histones and individual calf thymus histone H3, H2A, H2B and H4 were purchased from Roche. Recombinant H2A was from Upstate. Peptides were synthesized by the peptide synthesis service, Cancer Research UK. The sequences of the peptides are listed in Table S7.

### CO_2_ capture assay

Hydroxylation activity was determined radiochemically by measuring hydroxylation dependent release of [^14^C]CO_2_ as previously described [13]. Standard assay conditions comprised 40 µl reactions containing, 30 mM Hepes pH7.5, 90 µM 2-oxoglutarate, 10 µM [1-^14^C]2-oxoglutarate (PerkinElmer Life Sciences), 4 mM ascorbate, 250 µM (NH4)_2_Fe(SO_4_)_2_, purified Ofd2,Ofd2 H132A, alkbh1 or alkbh1 H228A and substrates as indicated. For each set of assays two stocks were made, one total volume 20 µl contained substrate, the second contained purified enzyme and all other reagents. Assays were started by the addition of 20 µl freshly prepared enzyme stock to the substrate stock. To recover [^14^C]CO_2_ a strip of Whatman 3 MM filter paper pre soaked in 30 mM calcium hydroxide was immediately inserted into the neck of the tube and the tube sealed. The assays were then incubated at 37°C for 60 min. Upon reaction completion, filter strips were removed, air dried, treated with scintillant and then counted for radioactivity in a scintillation counter. For screening the peptide library in Figure 2A the above conditions were adapted to a 96 well micro titre plate as previously described [29].

### Cell culture

Strains were grown at 30°C with shaking to exponential phase (OD = 0.5) in yeast extract media containing 2% glucose (YES) plus supplements (225 µg/ml each of histidine, leucine, adenine, lysine and uracil). Hypoxic (<1% O_2_) growth conditions were maintained using the AnaeroGen system (Oxoid), as per the manufacturer's instructions. For experiments using strains carrying the pREPNT81 vector, cells were first grown overnight in Edinburgh minimal media without leucine. The next day cells were transferred to YES media plus supplements and grown for 3 population doublings to OD = 0.5 before beginning hypoxia treatment.

### Microarray experiments

Exponentially growing cultures of wt (972) and Ofd2**Δ** (DL1003) were cultured in normal oxygen or hypoxic conditions for 90 min. Cells were harvested at 4°C and total RNA was isolated and purified using the RNeasy Mini Kit (Qiagen) in-conjunction with the RNase-Free DNase Set (Qiagen) to remove genomic DNA as per manufacturer's instructions. Five micrograms of total RNA from two independent experiments were then pooled and mRNA levels were quantified by Roche NimbleGen using the *S. pombe* 72K array service. Table S6 lists information on the target genes included on the array. Normalised gene expression values for each sample are provided in Table S1 as array duplicates. Log2 values, scatter plot graphing and calculations for thresholding were all carried out using Microsoft Excel. The microarray data (MIAME compliant) has been deposited at GEO (www.ncbi.nlm.nih.gov/geo/) accesion number GSE31236.

### Reverse transcriptase and quantitative PCR analysis

Total RNA was isolated as above. For each sample total RNA was processed to cDNA using the SuperScript III First-Strand synthesis system (Invitrogen) and oligo(dT) as per the manufacturer's instructions. Quantitaive PCR analysis was then carried out using the SYBR-green PCR mix (Applied Biosystems) with 20pmol primers on a ABI 7300 machine. The Pfaffl method was used to calculate changes in mRNA levels [30]. Primer pairs are listed in Table S8.

### Protein whole cell extracts

For each sample 2×10^7^ cells were resuspended in 250 µl lysis buffer (50 mM Tris-HCl pH8.0, 150 mM NaCl, 2 mM EDTA, 1% NP-40, 0.1% SDS and protease inhibitors) and boiled for 5 minutes. Then 250 µl of glass beads were added and cells broken by vortexing at 4°C.

### Chromatin Immunoprecipitation

Cells were cultured under normal oxygen or hypoxia conditions as stated, then DNA protein complexes were cross-linked by addition of formaldehyde to a concentration of 1% for 30 min at 4°C. Crosslinking was blocked with the addition of 100 mM glycine and cells were then washed once with ice cold PBS. Cells (1×10^8^) were resuspended in 500 µl FA lysis buffer (50 mM Hepes-KOH pH7.5, 140 mM NaCl, 1 mM EDTA, 1% Triton-X-100, 0.1% sodium deoxycholate, 0.1% SDS and protease inhibitors) and lysed by vortexing with glass beads at 4°C. Beads were removed and lysed extracts were sonicated with a Biorupter (Cosmo Bio) for 2×10 min (setting high: 30 s on, 30 s off) on ice. Sonicated extracts were clarified by centifugation at 13,000×g for 10 min at 4°C. Cleared lysates were diluted with 5 mls of RIPA buffer (50 mM Tris-HCl pH8.0, 150 mM NaCl, 2 mM EDTA, 1% NP-40, 0.5% sodium deoxycholate, 0.1% SDS and protease inhibitors) and 50 µl was removed to serve as input sample. Immunoprecipitation was carried out with 1 ml of lysate and 1 µg anti-flag monoclonal antibody (Sigma) or 1 µg mouse IgG (Sigma), overnight at 4°C. Next day pre-blocked protein A/G beads (GE healthcare) were added. After a 1 hr incubation at 4°C protein A/G bead complexes were pelleted and washed 3× with wash buffer (50 mM Tris-HCl pH8.0, 150 mM NaCl, 2 mM EDTA, 1% NP-40, 0.1% SDS) and 1× with wash buffer plus 500 mM NaCl. Bound DNA was eluted with 200 µl of elution buffer (100 mM NaHCO_3_, 1%SDS) for 15 min at 30°C. For input samples make up to 200 µl with elution buffer. Cross-linking was then reversed by incubating at 65°C for 5 hrs. The DNA was then purfied using the Qiagen PCR purification spin kit following the manufacturer's instructions. DNA amounts were then quantified by real-time PCR using SYBR-green PCR mix (Applied Biosystems) and ABI 7300 machine. For each amplicon a standard curve made up of tenfold dilutions of input DNA sample were used to calculate the amount of DNA in each corresponding immunopercipitated sample. Primer pairs are listed in Table S8.

### Mass spectrometry analysis of Ofd2-peptide reactions

Reactions were carried as described for CO_2_ capture assay except using non radioactive 2-oxoglutarate, 1ug of peptide and 1–10 µg of purified Ofd2. For mass spectrometry 0.5 µl of reaction samples were mixed with 1.5 µl of matrix solution (10 mg ml-1 -cyano-4-hydroxycinnamic acid in 50% (v/v) aqueous acetonitrile containing 0.1% (v/v) trifluoroacetic acid) and dried onto a MALDI target plate. Each sample spot was washed with 5 ul of 0.2% (v/v) heptafluorobutyric acid in water, dried, and analyzed on a Waters TofSpec2E MALDI mass spectrometer. Data were collected using a 500 MHz detector in reflectron mode. Calibration was three point between matrix ions and 1-31thioester peptide (1+ and 2+ charge states). The mass of 1–31thioester was checked independently as correct using internal standards of substance P and oxidized bovine insulin B chain. Calibration and m/z determination was carried out from centroid data. To rule out that components in the reaction were affecting the ability to detect mass changes, reaction samples were also cleaned up with C18 ZipTip (Millipore) as per manufacturers instructions and then analysed.

## Supporting Information

Figure S1 **Alkbh1 is a histone H2A dioxygenase.** Dioxygenase activity was evaluated with the CO_2_ capture assay using 1 µg of purified Alkbh1 with 25 µg calf thymus histones (cBH) or 5 µg of individual histones. Alkbh1 H228A ia an iron binding mutant. – indicate control reactions containing no substrate. Data is presented as mean from 2 replicates. Error bar equals 1 standard deviation.
(TIF)

Figure S2 **Substitutional analysis of peptide sequence C7.** Substitutional analysis of peptide sequence C7 with the CO_2_ capture assay using 1 µg of Ofd2 with 6 µg of peptide. Alanine and glycine substitutions are indicated in red. Data is from 2 replicates presented as a percent activity of captured CO_2_ relative to unmodified C7 peptide. Error bars equal 1 standard deviation.
(TIF)

Figure S3 **Hypoxia gene expression analysis of Ofd2 deletion strain.** Scatter plot analysis of fold change in gene expression after hypoxic treatment plotted as log2 values in wt against Ofd2Δstrain. Blue squares represent genes whose expression change was less than 2 fold in both strains. While green and red squares represent genes whose expression increased or decreased 2 fold or greater, in one or both strains. The gray line represents a threshold limit of 1.5 fold above and below values where the induced or repressed fold change for both wt and Ofd2 would be the same. Shaded box contain repressed genes that lie outside the threshold limit.
(TIF)

Table S1 **Array data set for all genes.**
(XLS)

Table S2 **Greater than 2 fold hypoxically upregulated genes in wt strain.**
(XLS)

Table S3 **Greater than 2 fold hypoxically down-regulated genes in wt strain.**
(XLS)

Table S4 **Greater than 2 fold hypoxically upregulated genes in Ofd2 deletion strain.**
(XLS)

Table S5 **Greater than 2 fold hypoxically down-regulated genes in Ofd2 deletion strain.**
(XLS)

Table S6 **Information on the microarray target genes.**
(XLS)

Table S7 **Peptide library.**
(DOC)

Table S8 **Quantitative PCR primer sets.**
(DOC)
