# Supplementary figures and images for: The S. pombe Histone H2A Dioxygenase Ofd2 Regulates Gene Expression during Hypoxia
Source: PLoS One. 2012 Jan 3;7(1):e29765. doi: 10.1371/journal.pone.0029765 (PMC3250473; doi:10.1371/journal.pone.0029765)

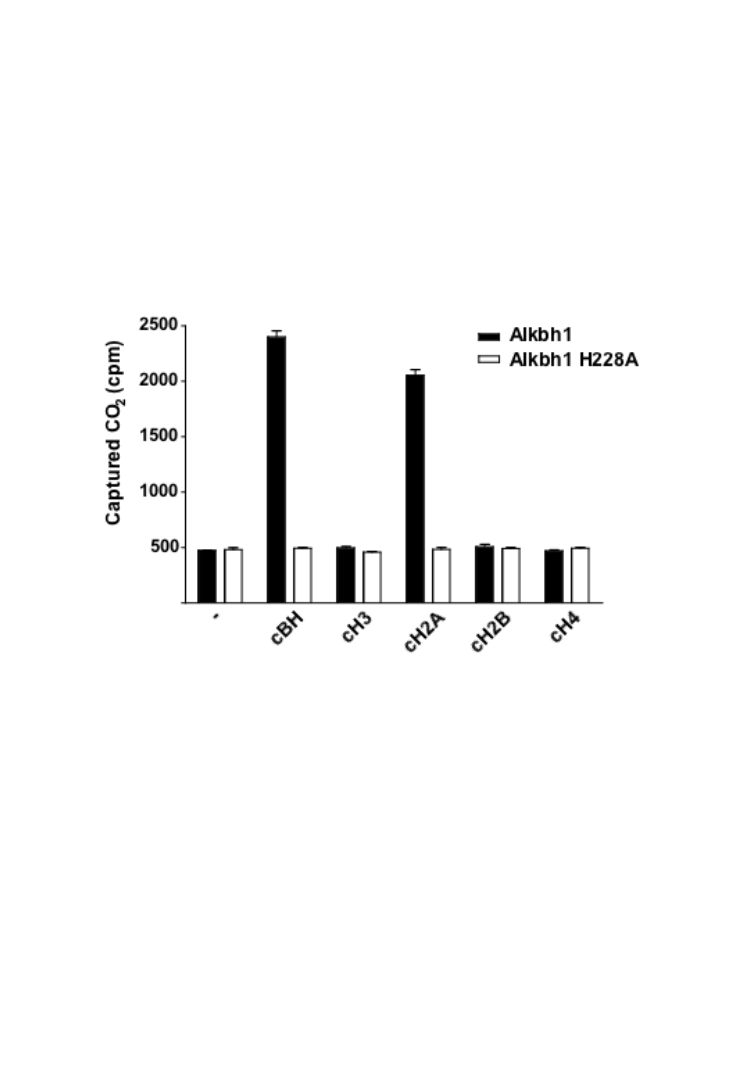

Supplement: Figure S1 — Alkbh1 is a histone H2A dioxygenase. Dioxygenase activity was evaluated with the CO2 capture assay using 1 µg of purified Alkbh1 with 25 µg calf thymus histones (cBH) or 5 µg of individual histones. Alkbh1 H228A ia an iron binding mutant. – indicate control reactions containing no substrate. Data is presented as mean from 2 replicates. Error bar equals 1 standard deviation. (TIF) [file pone.0029765.s001.tif]

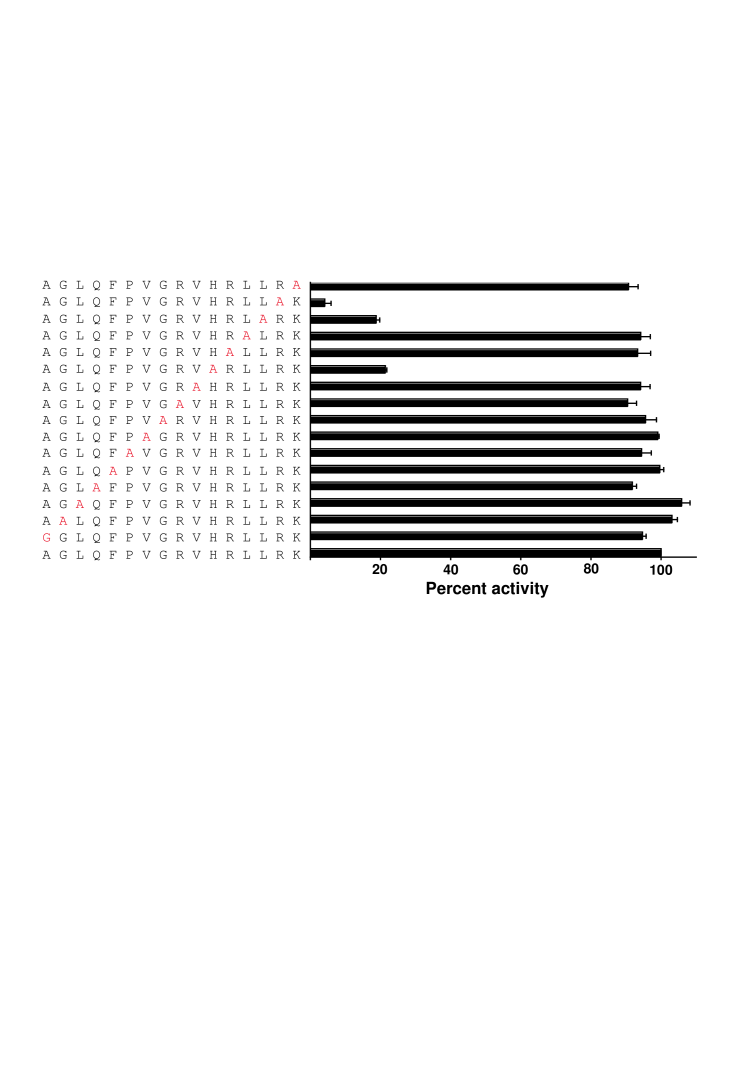

Supplement: Figure S2 — Substitutional analysis of peptide sequence C7. Substitutional analysis of peptide sequence C7 with the CO2 capture assay using 1 µg of Ofd2 with 6 µg of peptide. Alanine and glycine substitutions are indicated in red. Data is from 2 replicates presented as a percent activity of captured CO2 relative to unmodified C7 peptide. Error bars equal 1 standard deviation. (TIF) [file pone.0029765.s002.tif]

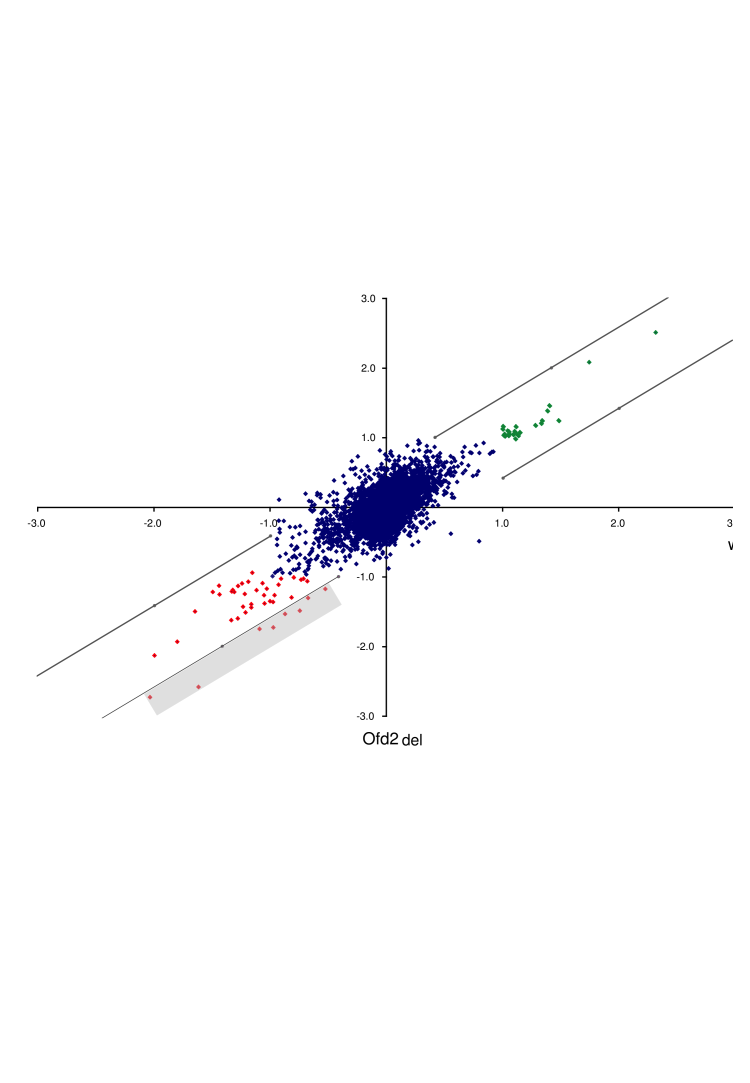

Supplement: Figure S3 — Hypoxia gene expression analysis of Ofd2 deletion strain. Scatter plot analysis of fold change in gene expression after hypoxic treatment plotted as log2 values in wt against Ofd2Δstrain. Blue squares represent genes whose expression change was less than 2 fold in both strains. While green and red squares represent genes whose expression increased or decreased 2 fold or greater, in one or both strains. The gray line represents a threshold limit of 1.5 fold above and below values where the induced or repressed fold change for both wt and Ofd2 would be the same. Shaded box contain repressed genes that lie outside the threshold limit. (TIF) [file pone.0029765.s003.tif]
