# Supplementary material for: The S. pombe Histone H2A Dioxygenase Ofd2 Regulates Gene Expression during Hypoxia
Source: PLoS One. 2012 Jan 3;7(1):e29765. doi: 10.1371/journal.pone.0029765 (PMC3250473; doi:10.1371/journal.pone.0029765)
Supplement: Table S7 — Peptide library. (DOC) [file pone.0029765.s010.doc]

No. Pos. Mol.Weight Sequence

1 A 1 1601.2 S-G-R-G-K-Q-G-G-K-A-R-A-K-A-K-T

2 A 2 1615.18 S-G-Rme-G-K-Q-G-G-K-A-R-A-K-A-K-T

3 A 3 1629.18 S-G-Rme2a-G-K-Q-G-G-K-A-R-A-K-A-K-T

4 A 4 1629.18 S-G-Rme2s-G-K-Q-G-G-K-A-R-A-K-A-K-T

5 A 5 1615.17 S-G-R-G-Kme-Q-G-G-K-A-R-A-K-A-K-T

6 A 6 1629.75 S-G-R-G-Kme2-Q-G-G-K-A-R-A-K-A-K-T

7 A 7 1643.17 S-G-R-G-Kme3-Q-G-G-K-A-R-A-K-A-K-T

8 A 8 1615.17 S-G-R-G-K-Q-G-G-Kme-A-R-A-K-A-K-T

9 A 9 1629.75 S-G-R-G-K-Q-G-G-Kme2-A-R-A-K-A-K-T

10 A10 1643.17 S-G-R-G-K-Q-G-G-Kme3-A-R-A-K-A-K-T

11 A11 1700.3 R-G-K-Q-G-G-K-A-R-A-K-A-K-T-R-S

12 A12 1714.28 R-G-K-Q-G-G-K-A-Rme-A-K-A-K-T-R-S

13 B 1 1728.28 R-G-K-Q-G-G-K-A-Rme2a-A-K-A-K-T-R-S

14 B 2 1728.28 R-G-K-Q-G-G-K-A-Rme2s-A-K-A-K-T-R-S

15 B 3 1730.3 K-Q-G-G-K-A-R-A-K-A-K-T-R-S-S-R

16 B 4 1744.27 K-Q-G-G-K-A-R-A-Kme-A-K-T-R-S-S-R

17 B 5 1758.85 K-Q-G-G-K-A-R-A-Kme2-A-K-T-R-S-S-R

18 B 6 1772.27 K-Q-G-G-K-A-R-A-Kme3-A-K-T-R-S-S-R

19 B 7 1602.2 G-G-K-A-R-A-K-A-K-T-R-S-S-R-A-G

20 B 8 1616.17 G-G-K-A-R-A-K-A-Kme-T-R-S-S-R-A-G

21 B 9 1630.75 G-G-K-A-R-A-K-A-Kme2-T-R-S-S-R-A-G

22 B10 1644.17 G-G-K-A-R-A-K-A-Kme3-T-R-S-S-R-A-G

23 B11 1729.3 K-A-R-A-K-A-K-T-R-S-S-R-A-G-L-Q

24 B12 1743.28 K-A-R-A-K-A-K-T-Rme-S-S-R-A-G-L-Q

25 C 1 1757.28 K-A-R-A-K-A-K-T-Rme2a-S-S-R-A-G-L-Q

26 C 2 1757.28 K-A-R-A-K-A-K-T-Rme2s-S-S-R-A-G-L-Q

27 C 3 1717.2 A-K-A-K-T-R-S-S-R-A-G-L-Q-F-P-V

28 C 4 1731.18 A-K-A-K-T-R-S-S-Rme-A-G-L-Q-F-P-V

29 C 5 1745.18 A-K-A-K-T-R-S-S-Rme2a-A-G-L-Q-F-P-V

30 C 6 1745.18 A-K-A-K-T-R-S-S-Rme2s-A-G-L-Q-F-P-V

31 C 7 1847.4 A-G-L-Q-F-P-V-G-R-V-H-R-L-L-R-K

32 C 8 1861.38 A-G-L-Q-F-P-V-G-Rme-V-H-R-L-L-R-K

33 C 9 1875.38 A-G-L-Q-F-P-V-G-Rme2a-V-H-R-L-L-R-K

34 C10 1875.38 A-G-L-Q-F-P-V-G-Rme2s-V-H-R-L-L-R-K

35 C11 1940.4 Q-F-P-V-G-R-V-H-R-L-L-R-K-G-N-Y

36 C12 1954.38 Q-F-P-V-G-R-V-H-Rme-L-L-R-K-G-N-Y

37 D 1 1968.38 Q-F-P-V-G-R-V-H-Rme2a-L-L-R-K-G-N-Y

38 D 2 1968.38 Q-F-P-V-G-R-V-H-Rme2s-L-L-R-K-G-N-Y

39 D 3 1940.4 V-G-R-V-H-R-L-L-R-K-G-N-Y-S-E-R

40 D 4 1954.38 V-G-R-V-H-R-L-L-Rme-K-G-N-Y-S-E-R

41 D 5 1968.38 V-G-R-V-H-R-L-L-Rme2a-K-G-N-Y-S-E-R

42 D 6 1968.38 V-G-R-V-H-R-L-L-Rme2s-K-G-N-Y-S-E-R

43 D 7 1940.4 G-R-V-H-R-L-L-R-K-G-N-Y-S-E-R-V

44 D 8 1954.37 G-R-V-H-R-L-L-R-Kme-G-N-Y-S-E-R-V

45 D 9 1968.95 G-R-V-H-R-L-L-R-Kme2-G-N-Y-S-E-R-V

46 D10 1982.37 G-R-V-H-R-L-L-R-Kme3-G-N-Y-S-E-R-V

47 D11 1674.1 L-R-K-G-N-Y-S-E-R-V-G-A-G-A-P-V

48 D12 1688.08 L-R-K-G-N-Y-S-E-Rme-V-G-A-G-A-P-V

49 E 1 1702.08 L-R-K-G-N-Y-S-E-Rme2a-V-G-A-G-A-P-V

50 E 2 1702.08 L-R-K-G-N-Y-S-E-Rme2s-V-G-A-G-A-P-V

51 E 3 1770.3 L-E-L-A-G-N-A-A-R-D-N-K-K-T-R-I

52 E 4 1784.28 L-E-L-A-G-N-A-A-Rme-D-N-K-K-T-R-I

53 E 5 1798.28 L-E-L-A-G-N-A-A-Rme2a-D-N-K-K-T-R-I

54 E 6 1798.28 L-E-L-A-G-N-A-A-Rme2s-D-N-K-K-T-R-I

55 E 7 1781.3 A-G-N-A-A-R-D-N-K-K-T-R-I-I-P-R

56 E 8 1795.27 A-G-N-A-A-R-D-N-Kme-K-T-R-I-I-P-R

57 E 9 1809.85 A-G-N-A-A-R-D-N-Kme2-K-T-R-I-I-P-R

58 E10 1823.27 A-G-N-A-A-R-D-N-Kme3-K-T-R-I-I-P-R

59 E11 1847.3 G-N-A-A-R-D-N-K-K-T-R-I-I-P-R-H

60 E12 1861.27 G-N-A-A-R-D-N-K-Kme-T-R-I-I-P-R-H

61 F 1 1875.85 G-N-A-A-R-D-N-K-Kme2-T-R-I-I-P-R-H

62 F 2 1889.27 G-N-A-A-R-D-N-K-Kme3-T-R-I-I-P-R-H

63 F 3 1917.4 A-A-R-D-N-K-K-T-R-I-I-P-R-H-L-Q

64 F 4 1931.38 A-A-R-D-N-K-K-T-Rme-I-I-P-R-H-L-Q

65 F 5 1945.38 A-A-R-D-N-K-K-T-Rme2a-I-I-P-R-H-L-Q

66 F 6 1945.38 A-A-R-D-N-K-K-T-Rme2s-I-I-P-R-H-L-Q

67 F 7 1899.6 K-T-R-I-I-L-A-P-R-H-L-Q-L-A-I-R

68 F 8 1913.58 K-T-R-I-I-L-A-P-Rme-H-L-Q-L-A-I-R

69 F 9 1927.58 K-T-R-I-I-L-A-P-Rme2a-H-L-Q-L-A-I-R

70 F10 1927.58 K-T-R-I-I-L-A-P-Rme2s-H-L-Q-L-A-I-R

71 F11 1946.3 P-R-H-L-Q-L-A-I-R-N-D-E-E-L-N-K

72 F12 1960.28 P-R-H-L-Q-L-A-I-Rme-N-D-E-E-L-N-K

73 G 1 1974.28 P-R-H-L-Q-L-A-I-Rme2a-N-D-E-E-L-N-K

74 G 2 1974.28 P-R-H-L-Q-L-A-I-Rme2s-N-D-E-E-L-N-K

75 G 3 1855.4 I-R-N-D-E-E-L-N-K-L-L-G-K-V-T-I

76 G 4 1869.37 I-R-N-D-E-E-L-N-Kme-L-L-G-K-V-T-I

77 G 5 1883.95 I-R-N-D-E-E-L-N-Kme2-L-L-G-K-V-T-I

78 G 6 1897.37 I-R-N-D-E-E-L-N-Kme3-L-L-G-K-V-T-I

79 G 7 1654.3 E-L-L-N-K-L-L-G-K-V-T-I-A-Q-G-G

80 G 8 1668.27 E-L-L-N-K-L-L-G-Kme-V-T-I-A-Q-G-G

81 G 9 1682.85 E-L-L-N-K-L-L-G-Kme2-V-T-I-A-Q-G-G

82 G10 1696.27 E-L-L-N-K-L-L-G-Kme3-V-T-I-A-Q-G-G

83 G11 1843.2 N-I-Q-A-V-L-L-P-K-K-T-E-S-H-H-K

84 G12 1857.17 N-I-Q-A-V-L-L-P-Kme-K-T-E-S-H-H-K

85 H 1 1871.75 N-I-Q-A-V-L-L-P-Kme2-K-T-E-S-H-H-K

86 H 2 1885.17 N-I-Q-A-V-L-L-P-Kme3-K-T-E-S-H-H-K

87 H 3 1800.2 I-Q-A-V-L-L-P-K-K-T-E-S-H-H-K-A

88 H 4 1814.17 I-Q-A-V-L-L-P-K-Kme-T-E-S-H-H-K-A

89 H 5 1828.75 I-Q-A-V-L-L-P-K-Kme2-T-E-S-H-H-K-A

90 H 6 1842.17 I-Q-A-V-L-L-P-K-Kme3-T-E-S-H-H-K-A

91 H 7 1744.2 A-V-L-L-P-K-T-E-S-H-H-K-A-K-G-K

92 H 8 1758.17 A-V-L-L-P-K-T-E-S-H-H-Kme-A-K-G-K

93 H 9 1772.75 A-V-L-L-P-K-T-E-S-H-H-Kme2-A-K-G-K

94 H10 1786.17 A-V-L-L-P-K-T-E-S-H-H-Kme3-A-K-G-K

Mono (Kme1), di (Kme2) and tri (Kme3) methyl lysine

Mono (Rme1) symmetric di (Rme2s) and asymmetric di (Rme2a) methyl arginine
