# Supplementary material for: The S. pombe Histone H2A Dioxygenase Ofd2 Regulates Gene Expression during Hypoxia
Source: PLoS One. 2012 Jan 3;7(1):e29765. doi: 10.1371/journal.pone.0029765 (PMC3250473; doi:10.1371/journal.pone.0029765)
Supplement: Table S8 — Quantitative PCR primer sets. (DOC) [file pone.0029765.s011.doc]

| Gene |  | Forward | Reverse |
| --- | --- | --- | --- |
| act1 |  | 5’tgc tcc tcc tga gcg taa ata | 5’ccg ctc tca tca tac tct tgc |
|  |  |  |  |
| *cyc1* | A1 | 5’tgc gat caa atg ctt ctt tg | 5’cat ggg aat aca cca ttg ga |
|  | A2 | 5’cgg aga gct cat gaa cca at | 5’cac agc gaa gtt agt gag aag atg |
|  | A3 | 5’tcg ctg tga att tgt ggt tt | 5’gaa gca ccc ttc ttt tcg tc |
|  | A4^*^ | 5’cca atc gcg ata agg gta tt | 5’gtt acg atc agc cgg ttt ct |
|  |  |  |  |
| *qcr8* | A1 | 5’agc tct gct gca tca aac tg | 5’tgt att aca ccg tcc cga ag |
|  | A2 | 5’gga agt ccg ttt gag caa ga | 5’gcg ggt ctg cta gac caa ta |
|  | A3 | 5’gac ccg cac act tac cct ta | 5’aag ttt ttc ctc cag cag ca |
|  | A4^*^ | 5’cct ttc ccc att tca aca ga | 5’ctc ctt ccc cca aca gta aa |
|  |  |  |  |
| *SPAC3A11.07* | A1 | 5’ttc ctg ccc ata aac acc tc | 5’cgc taa atg tcc caa ttt cc |
|  | A2 | 5’ccc tgt ccg act ttg aca ac | 5’cgc aga taa gcg tcg att ta |
|  | A3 | 5’ctc gaa gtt ttc tag tag ttg ctt g | 5’gcc gat aaa gct aag gga ga |
|  | A4 | 5’tgt cta ccg ttt ccg tca tc | 5’aat aga agt ggc acc cca ac |
|  | A5^*^ | 5’gtg aaa tgg ctg att tca ttg | 5’taa ctt ggc gct aaa cat gg |
|  |  |  |  |
| *erg3* | A1 | 5’ctt gtt gaa tag cga gcc | 5’ata ggg cag atc gga aag |
|  | A2 | 5’cca tca att cga cac gaa ga | 5’aat tac ggc caa ttc acc ac |
|  | A3^*^ | 5’atc tcc aac aac ccc cat cat | 5’ttt gtc aaa cca tgc ctc at |
|  |  |  |  |
| *hem13* |  | 5’caa gat ggc aac gtt ttt ga | 5’gga agc tct ttg gca gta cg |

^*^ Primer pairs used for both ChIP and RT-qPCR
